# Improving the Oxygen Evolution Reaction Performance of Ternary Layered Double Hydroxides by Tuning All Three Cations’ Electronic Structures

**DOI:** 10.3390/nano15030177

**Published:** 2025-01-23

**Authors:** Gayi Nyongombe, Malik Maaza, Mohamed Siaj, Simon Dhlamini

**Affiliations:** 1 Department of Physics, School of Science, CSET, University of South Africa, Private Bag X6, Florida, 1710, Science Campus, Christiaan de Wet and Pioneer Avenue, Florida Park, Johannesburg 1710, South Africa; 2 NanoQam Center, Department of Chemistry, University of Quebec, Montreal, QC H3C3P8, Canada; siaj.mohamed@uqam.ca; 3 UNESCO-UNISA Africa Chair in Nanoscience/Nanotechnology, College of Graduate Studies, University of South Africa, Muckleneuk Ridge, Pretoria P.O. Box 392, South Africa; maazam@unisa.ac.za

**Keywords:** layered double hydroxides, ternary, sodium dodecyl sulfate, oxygen evolution reaction, electrocatalyst, electrochemical

## Abstract

The pursuit of efficient and sustainable hydrogen production is essential in the fight against climate change. One important method for achieving this is the electrolysis of water, particularly through the oxygen evolution reaction (OER). Recent studies indicate that trimetallic layered double hydroxides (LDHs) can enhance OER performance compared to bimetallic LDHs. This improvement occurs because the third cation alters the electronic structures of the other two cations, thereby increasing the intermediates’ binding energies and enhancing electrical conductivity. This study proposes an approach enabling the modulation of the electronic structures of all three cations involved in the synthesis of the trimetallic LDHs. It suggested intercalating sodium dodecyl sulfate (SDS) into the interlayer of the trimetallic NiFe-La-LDH. A successful intercalation of SDS has been confirmed through the XRD, FT-IR, EDS, and XPS. This has expanded the interlayer distance which was beneficial for the electrical conductivity. Furthermore, SDS generated sulphur, which modulated the electronic structures of all three cations enriching the active sites and improving electrical conductivity and OER performance compared to its counterparts. This approach is beneficial: 1. The interlayer can be further enlarged by using different doping ratios of SDS. 2. Sulphur can enrich the active sites and improve the OER performance.

## 1. Introduction

In the current event of climate change, producing clean energy like hydrogen is a vital act in decreasing carbon consumption, particularly in relation to the usage of fossil fuels, which are the principal contributors to environmental challenges such as global warming and air pollution [1,2,3]. Electrocatalytic water splitting (EWS) is considered a more environmentally friendly method for producing pure hydrogen, as it generates hydrogen from water without emitting carbon dioxide (CO_2_). EWS consists of two half-reactions: the oxygen evolution reaction (OER) occurring at the anode and the hydrogen evolution reaction (HER) occurring at the cathode [4,5,6]. Comparing both half-reactions, OER is considered to impede the industrial application of electrocatalytic water splitting (EWS) due to its sluggish kinetics triggered by its four proton-coupled electron transfer processes that promote a high overpotential for EWS [5]. Generally, the performance of OER relies on the physicochemical properties of the electrocatalyst used. Hence, countless efforts have been dedicated to improving the performance of OER using electrocatalysts with excellent catalytic properties [2]. Consequently, several electrocatalysts such as β-Nis@Ni(OH)_2_, NiFe/NiCo_2_O_4_, NixFe1−xSe2, and layered double hydroxides (LDHs) have been reported to increase the catalytic efficiency of OER [2,7,8,9].

Layered double hydroxides (LDHs) are 2D materials also known as ionic lamellar compounds with [M(II)_1−x_ M(III)x (OH)_2_] (Y^n−^)_x/n_·yH_2_O as a general formula; where M(II) and M(III) are divalent and trivalent metal cations, and Y^n−^ designates the anion between the layers [10]. Technically, their crystalline structure is made of positively charged stacking layers composed of divalent and trivalent metal cations; meanwhile, the negatively charged anion and water molecules reside in the interlayer [11]. To date, a wide range of electrochemical applications for LDHs have been extensively documented in the literature. These applications primarily focus on areas such as photoluminescence [12], drug delivery [13], sensors [14], cosmetics [15], supercapacitors [16], lithium batteries [17], and electrocatalysts for HER and OER [2]. Compared to other electrocatalysts for OER, LDHs offer numerous advantages, including easy synthesis with large-scale production ability which is essential for industrial applications [16,18]. Generally, they are made of abundant, low-cost, and environmentally friendly precursors that make their usage safe and convenient [18]. LDHs have a unique structure that allows a consistent distribution of various metal cations within the brucite layer. They also possess surface hydroxyl groups and can be easily tuned. Additionally, the presence of intercalated anions in their interlayer space enables good chemical stability and the capability to accommodate a wide range of anions, including inorganic, organic, biomolecules, and even genes [16]. Bimetallic LDHs consist of two metal cations and are the most frequently reported for the OER, with NiV-LDHs, NiCo-LDHs, and NiFe-LDHs being particularly prominent [18]. However, bimetallic LDHs generally suffer from poor electrical conductivity and fewer accessible active sites, which restrict their application in real energy devices [19]. To tackle these challenges, significant efforts have been made to incorporate a third metal cation into bimetallic LDHs to form trimetallic LDHs also known as ternary LDHs. This strategy modulates the cations’ electronic structure and charge density, increasing the intermediates’ binding energies and improving the electrical conductivity. It also enriches the active sites and shortens the band gap, favoring an easier transfer of electrons and finally enhancing the OER performance [18,19,20,21,22]. Recently, Zhou et al. improved the OER performance by incorporating a third metal cation into NiFe-LDHs. This modification increased the electron density in Ni^2^⁺ and Fe^3^⁺ by facilitating the partial transfer of electrons to their surroundings within the hydroxide matrix [23]. Following this, Jiang et al. reported an enhancement in the OER performance of Ni_3_Fe_0.5_V_0.5_-LDHs, attributing it to the increased electron density. The authors concluded that the improvement in the OER performance was driven by the presence of the third metal cation, which influenced the local coordination environment of both Ni and Fe through mutual interactions [24]. Furthermore, additional studies confirmed that introducing a third metal cation can generate various defects and vacancies, which serve as active sites for both cations. These active sites facilitate the adsorption, desorption, and transfer of oxygen-based intermediates during the OER process [25,26]. Though the ternary LDHs approach has proven effective in enhancing the OER performance, it is crucial to acknowledge that introducing a third cation only modulates the electronic structures of the two other cations involved in the LDHs synthesis even though the final product is a trimetallic LDHs.

This work presents a promising approach that enables the modulation of the electronic structures of all three cations involved in the synthesis of ternary LDHs. The proposed approach suggests intercalating Sodium Dodecyl Sulfate (SDS) into the interlayer space of the ternary LDHs. Then, apart from enlarging the interlayer distance which contributes to improving the electrical conductivity [27,28,29], SDS also generates sulfur which modulates the electronic structures of all three cations, enriches the active sites, improves the kinetics, ameliorates the electron transfer, increases the specific surface area, improves the electrocatalytic properties, and finally yields a good OER performance. Technically, SDS was intercalated into NiFe-La-LDHs expanding its interlayer distance which was advantageous for the electrical conductivity. More importantly, sulfur produced by SDS modulated the electronic structures of nickel (Ni), iron (Fe), and lanthanum (La), resulting in improved OER performance compared to electrocatalysts without SDS. Furthermore, the specific surface area increased by 2.5 times compared to samples without SDS. To our knowledge, this study is the first of its kind on this topic. Further details about the findings are provided below.

## 2. Materials and Methods

### 2.1. Reagents

Nickel nitrate hexahydrate (Ni (NO_3_)_2_•6H_2_O), Iron nitrate nonahydrate (Fe (NO_3_)_3_•9H_2_O), Lanthanum nitrate hexahydrate (La (NO_3_)_3_•6H_2_O), Sodium Dodecyl Sulfate (SDS), Ammonium fluoride (NH_4_F), and Urea (CH_4_N_2_O) were purchased from Sigma-Aldrich (Merck KGaA Inc., Darmstadt, Germany) and used as received. Deionized (DI) water collected from a water purification machine (Milli-Qs) was used in the experiment and to wash the samples.

### 2.2. Methods

**NiFe-LDH** was synthesized via the hydrothermal method. Typically, 1.46 gr of (Ni (NO_3_)_2_•6H_2_O) and 1.21 gr of (Fe (NO_3_)_3_•9H_2_O) were dissolved into 80 mL of deionized (DI) water and sonicated for 30 min. Afterwards, 0.15 gr of NH_4_F and 1 gr of urea were added into the above solution and sonicated for another 10 min. Thereafter, the final solution was transferred into a 100 mL Teflon-lined stainless-steel autoclave and aged for 6 h at 100 °C in an oven. Subsequently, the autoclave was permitted to cool down at ambient temperature; then, the precipitates were collected and washed several times with DI water and ethanol and dried at 80 °C for 12 h. Secondly, 0.25 gr of (La (NO_3_)_3_•6H_2_O) was dispersed into 80 mL of DI water alongside 1.46 gr of (Ni (NO_3_)_2_•6H_2_O) and 1.21 gr of (Fe (NO_3_)_3_•9H_2_O) and followed the synthesis path disclosed earlier. Then, the as-obtained sample was named **NiFe-La-LDH**. Thirdly, 0.25 gr of (La (NO_3_)_3_•6H_2_O), 1.46 gr of (Ni (NO_3_)_2_•6H_2_O), and 1.21 gr of (Fe (NO_3_)_3_•9H_2_O) were all dissolved into 60 mL of DI water and sonicated for 30 min. Thereafter, 0.15 gr of NH_4_F and 1 gr of urea were added into the above solution and sonicated for another 10 min (solution A). On the other side, 0.50 gr of Sodium Dodecyl Sulfate (SDS) was dispersed into 20 mL of DI water (solution B). Then, solution B was poured into solution A under vigorous stirring for 1 h and followed the same synthesis route as its counterparts. Thus, the as-prepared sample was titled **NiFe-La-SDS-LDH**.

### 2.3. Characterization Section

The crystallinity of the obtained samples was characterized by X-ray crystallography (XRD) using a diffractometer (Bruker D8 Advance, Bruker Corporation, Karlsruhe, Germany) with Cu-Ka radiation (λ = 1.54182 Å). The Fourier transform infrared (FTIR) spectra were recorded using the Fourier transform infrared spectroscopy (Thermo Scientific Inc., Waltham, MA, USA, Nicolet 6700/smart iTR). A Joel microscope (JSM7600F, JEOL Ltd., Peabody, MA, USA) operated at 10 kV accelerating voltage served for the scanning electron microscope tests (SEM), and the morphologies of all samples were captured. Meanwhile, a Joel JEM-2100F microscope (JEOL Ltd., Peabody, MA, USA) served to perform the energy-dispersive X-ray spectroscopy (EDS) measurements. For the Brunauer-Emmett-Teller (BET) measurements, a TMAX-BSD-PM2 BET (Xiamen Tmax Battery Equipments Limited, Xiamen, China) was used to collect the specific surface areas and textural properties. The X-ray photoelectron spectroscopy (XPS) data were recorded using ESCALAB 250Xi (source: Mono Al Kα 1486.68 eV, Thermo Scientific Inc., Waltham, MA, USA); while all spectra were analyzed using avantage v6.5 and the high-resolution peak components were fitted using symmetrical Lorentzian/Gaussian peak.

### 2.4. Electrocatalytic Measurements

The electrochemical tests were performed in 1 M KOH solution using a BioLogic SP-300 with a three-electrode system (BioLogic Science Instruments, Seyssinet-Pariset, France). Ag/AgCl was used as a reference electrode, Pt wire as a counter electrode and the as-obtained samples as working electrodes. The working electrodes were all prepared by mixing the as-obtained materials with 80 μL of Nafion solution (5 wt%) and 150 μL of water-ethanol solution with a volume ratio of 4:1, and the mixture was then sonicated for 30 min. Afterwards, the obtained ink was drop-casted onto cleaned nickel foam (1 cm × 1 cm) and dried at 60 °C for 1 h. The Cyclic voltammetry (CV) curves were recorded at a potential range of 0–0.6 V at different scan rates from 10 to 100 mV/s. The linear sweep voltammetry (LSV) measurement was carried between 0.0 and 1.5 V at the scanning rate of 10 mV/s for OER. The stability test was carried out via the chronoamperometry technique for NiFe-La-LDH and NiFe-La-SDS-LDH electrocatalysts using potentials recorded at the onset points of their LSV curves. The electrochemical Impedance Spectroscopy (EIS) was also carried out at an amplitude of 10 mV in a frequency ranging from 0.1 Hz to 100 KHz.

## 3. Results

**Figure 1:**
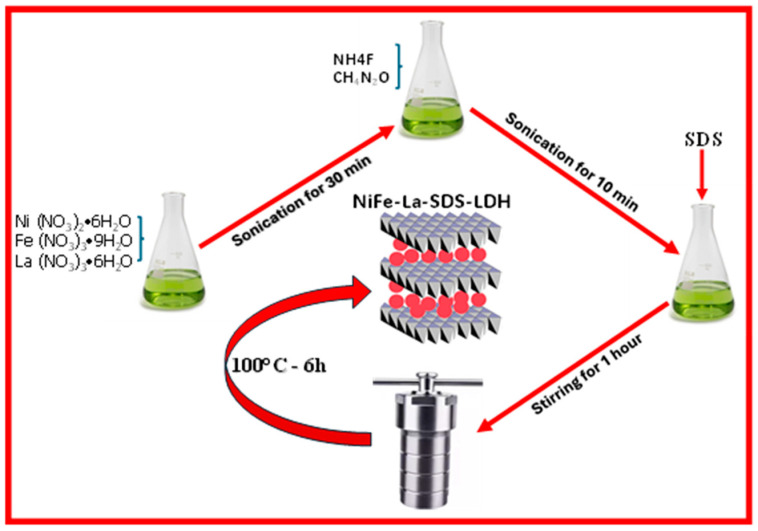
Schematic illustration of the synthesis route.

**Figure 2:**
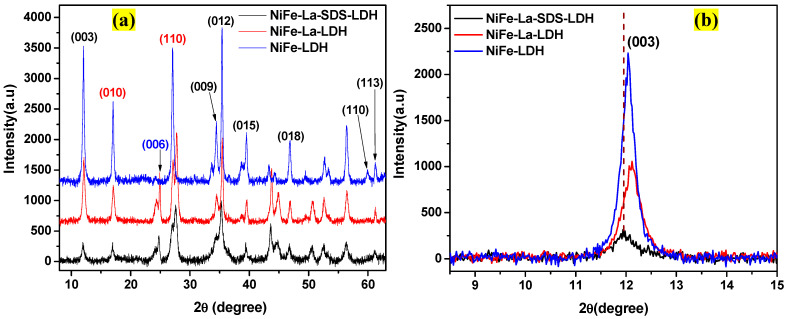
(**a**) XRD patterns for NiFe-LDH, NiFe-La-LDH, and NiFe-La-SDS-LDH; (**b**) Comparison of diffraction peaks indexed to (003) for NiFe-LDH, NiFe-La-LDH, and NiFe-La-SDS-LDH.

The synthesis route is displayed in Figure 1. Meanwhile, Figure 2a shows the XRD patterns of NiFe-LDH, NiFe-La-LDH, and NiFe-La-SDS-LDH. Diffraction peaks ascribed to the plane of hydrotalcite structure were observed at 12.06°, 34.3°, 35.42°, 39.56°, 46.7°, 59.88°, and 61.2°, and indexed to (003), (009), (012), (015), (018), (110), and (113), and were recorded for all the samples [26,30,31]. Generally, diffraction peaks located at lower angles ranging from 10° to 35° confirm the formation of LDHs structure. Hence, diffraction peaks indexed to (003), (009), and (012) depicted on the XRD patterns of all the samples indicate a successful formation of LDHs [10]. Subsequently, it was observed that after adding La into NiFe-LDH, a diffraction peak located at 24.86° indexed to (006) appeared in the XRD patterns of NiFe-La-LDH and NiFe-La-SDS-LDH. Technically, diffraction peaks indexed to (003), (006), and (009) reveal the nature of the environment within the interlayer; it also defines the distance between interlamellar, also known as the lattice constant “c” [30]. Hence, the diffraction peak positioned at 24.86°, indexed to (006), observed after adding La into NiFe-LDH, could indicate a change in the lattice constant “c” induced by La. Furthermore, shifts in the position of many diffraction peaks were also recorded after incorporating La; this discloses a change in the interlayer environment. More importantly, it was noticed that the diffraction peak indexed to (110) completely disappeared when La was introduced. Generally, diffraction peaks indexed to (110) and (113) inform about the lattice constant “a”, which refers to the distance between cations within lamellar layers [10]. Thus, the occurred situation could be attributed to a change in the lattice constant “a” due to the addition of a new cation which is La. Subsequently, two peaks located at 17.82° and 27.0° indexed to (010) and (110) were also depicted and assigned to planes of FeOOH [26]. Figure 2b compares the diffraction peaks indexed to (003) for all the samples. As it can be seen, the diffraction peak indexed to (003) for NiFe-La-SDS-LDH displayed a shift towards lower angles compared to those of its counterparts indicating a successful intercalation of SDS into the interlayer [10,32]. This also suggests an increase in the interlayer distance, which could improve the electrical conductivity [31].

**Figure 3:**
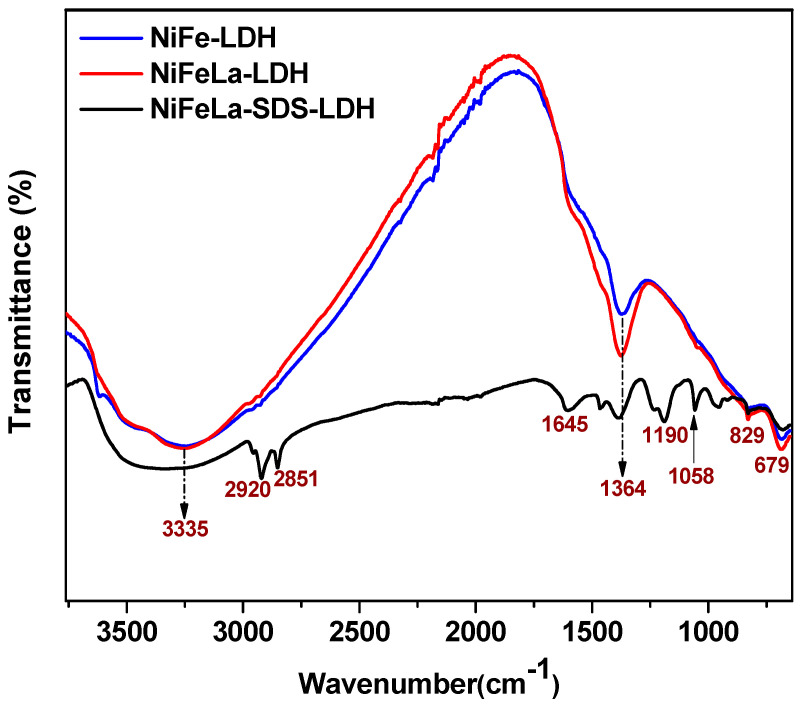
FT-IR spectra for NiFe-LDH, NiFe-La-LDH, and NiFe-La-SDS-LDH.

Figure 3 displays the FT-IR spectra of NiFe-LDH, NiFe-La-LDH, and NiFe-La-SDS-LDH. As it can be noticed, NiFe-LDH and NiFe-La-LDH displayed similar FT-IR spectra exhibiting broad bands at 3335 cm^−1^ assigned to O-H stretching vibrations related to water molecules at the brucite-like layer and interlayer. Followed by bands at 1364 cm^−1^ ascribed to the vibrations of the intercalated ${CO}_{3}^{2-}$ probably generated by the atmospheric CO_2_ [33,34]. It is worth noting that the band positioned at 1364 cm^−1^ for NiFe-La-LDH was more prominent compared to that of NiFe-LDH, indicating a difference in the nature of interactions of the intermolecular strength such as hydrogen bonding in each sample and the influence of affected molecules of water in their chemical environments due to the presence of La. This is in accordance with the XRD results discussed earlier [11]. Lastly, two bands located at 829 and 678 cm^−1^ were depicted and assigned to metal-oxygen (M-O) stretching and bending vibrations in the brucite-like lattice, indicating the presence of metal ions in the composite [16]. In contrast, the FT-IR spectrum of NiFe-La-SDS-LDH displayed vibrational bands similar to those of NiFe-LDH and NiFe-La-LDH, alongside other new bands located at 2920 and 2851 cm^−1^ attributed to the alkyl chains of dodecyl sulfate occurring in the symmetric and antisymmetric ${-CH}_{2}$ stretching modes [35]. Followed by 1190 and 1058 cm^−1^ assigned to the S=O antisymmetric stretching mode of SDS [27]. This indicates a successful intercalation of SDS in the interlayer of NiFe-La-SDS-LDH supporting the XRD results discussed earlier. Finally, the band at 1645 cm^−1^ was ascribed to vibrations of adhering water molecules, likely due to the altered water molecules in the chemical environments caused by a change in the intermolecular strength [36].

**Figure 4:**
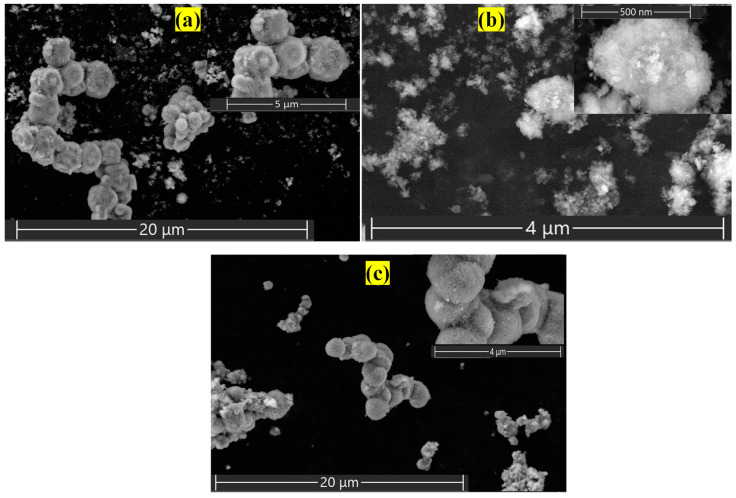
FE-SEM micrograph of: (**a**) NiFe-LDH, (**b**) NiFe-La-LDH, (**c**) NiFe-La-SDS-LDH.

**Figure 5:**
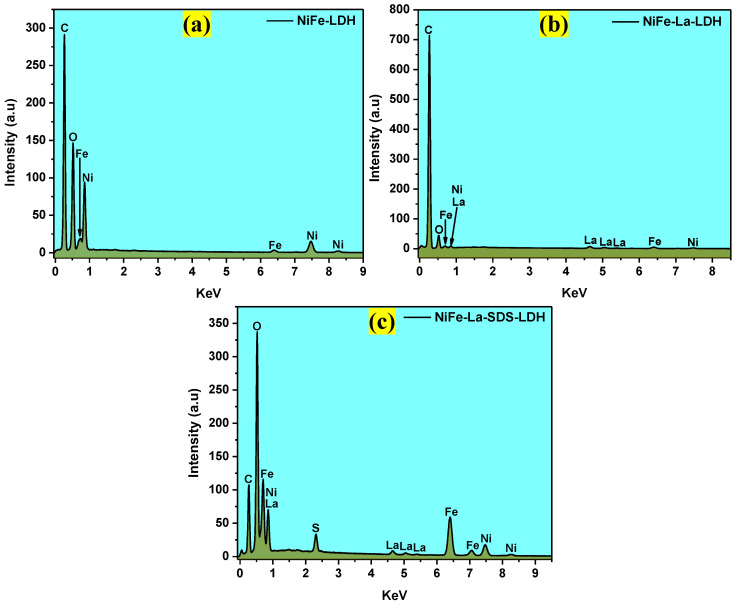
ESD spectra for: (**a**) NiFe-LDH, (**b**) NiFe-La-LDH, (**c**) NiFe-La-SDS-LDH.

The morphologies of all the samples were captured by SEM. Figure 4a–c displays the SEM images of NiFe-LDH, NiFe-La-LDH, and NiFe-La-SDS-LDH, respectively. Aggregated ball-like structures were observed for NiFe-LDH (Figure 4a) and NiFe-La-SDS-LDH (Figure 4c). While NiFe-La-LDH (Figure 4b) showed an almost spherical shape alongside scattered nano-sheets. Subsequently, the EDS results revealed the chemical elements present in all the obtained samples. Figure 5a–c show the EDS spectra of NiFe-LDH, NiFe-La-LDH, and NiFe-La-SDS-LDH. The presence of Nickel, Iron, and Oxygen were depicted in NiFe-LDH. While Nickel, Iron, Lanthanum, and Oxygen were noticed in NiFe-La-LDH. Lastly, Nickel, Iron, Lanthanum, Sulphur, and Oxygen were observed in NiFe-La-SDS-LDH. The presence of sulphur noticed in NiFe-La-SDS-LDH could probably be generated by SDS. Moreover, Figure S1a–c show mapping images for all the samples disclosing the homogeneous distribution of all expected chemical elements. The observed carbon was the result of SEM tape used during sample preparation.

**Figure 6:**
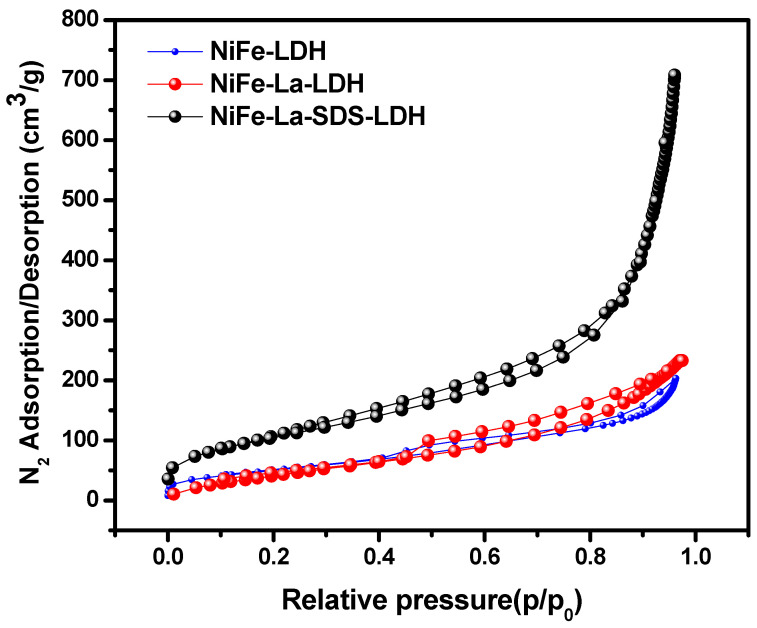
N_2_-sorption isotherms of: NiFe-LDH, NiFe-La-LDH, and NiFe-La-SDS-LDH.

To understand the induced effect of SDS on the specific surface areas and textural properties, N_2_ adsorption-desorption analysis was conducted. Figure 6 shows the nitrogen adsorption-desorption isotherm of NiFe-LDH, NiFe-La-LDH, and NiFe-La-SDS-LDH displaying hysteresis loops of type IV isotherm indicating mesoporous structures for all the samples [37]. The textural properties of NiFe-LDH, NiFe-La-LDH, and NiFe-La-SDS-LDH are disclosed in Table 1. As it can be observed, NiFe-La-SDS-LDH exhibited a high surface area, bigger pore sizes, as well as bigger pore volume compared to its counterparts. Followed by NiFe-La-LDH which recorded a high surface area and bigger pore volume compared to NiFe-LDH. However, its pores sizes are smaller than those of NiFe-LDH. Generally, the nature of the pore volume is the result of the formation process and cations that constitute the layer. Whereas, the size of pores is the result of the preparation process as well as the relationship between the lamellar [11,30]. This shows that either La or SDS have affected these properties accordingly.

**Figure 7:**
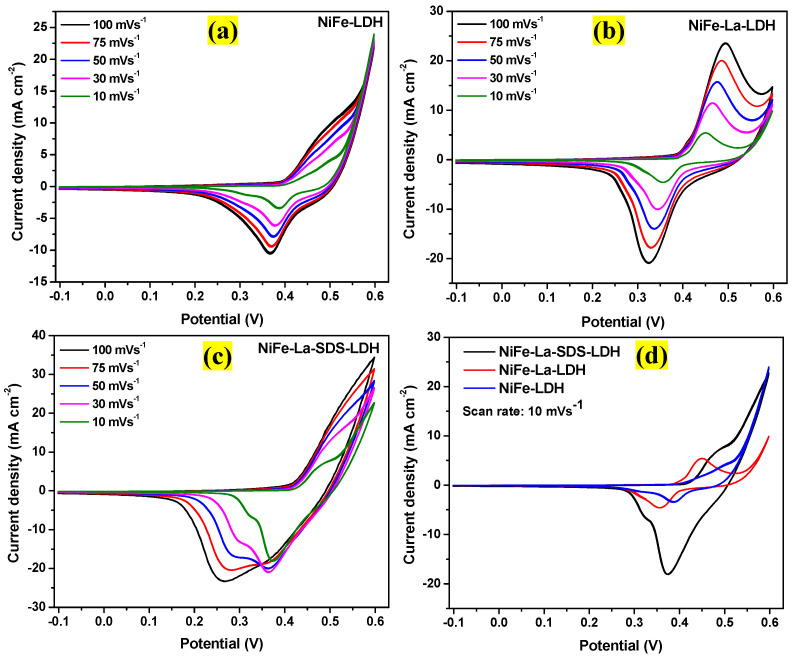
(**a**–**c**) CV curves at various scan rates (10–100 mVs^−1^), and (**d**) Comparative CV curves at a scan rate of 10 mVs^−1^ for NiFe-LDH, NiFe-La-LDH, and NiFe-La-SDS-LDH.

The electrocatalytic performance of NiFe-LDH, NiFe-La-LDH, and NiFe-La-SDS-LDH electrocatalysts were studied in a three-electrode system using Pt wire and Ag/AgCl as counter and reference electrodes, respectively. All the as-obtained samples served as electrocatalysts and were prepared as described in the experimental section. All the electrochemical measurements were carried out in 1 M KOH electrolyte. Figure 7a–c showed the cyclic voltammetry (CV) curves for NiFe-LDH, NiFe-La-LDH, and NiFe-La-SDS-LDH electrocatalysts at different scan rates ranging from 10–100 mVs^−1^. As it can be noticed, all the electrocatalysts displayed prominent oxidation and reduction peaks that emerged due to the insertion and desertion of ions from the electrolyte during the anodic and cathodic reactions [38]. Meanwhile, Figure 7d compares the CV curves of NiFe-LDH, NiFe-La-LDH, and NiFe-La-SDS-LDH electrocatalysts at the scan rate of 10 mVs^−1^. It is evident that the integrated CV area of NiFe-La-SDS-LDH electrocatalyst is larger compared to its counterparts, along with the high current-voltage response. This could indicate good electrical conductivity and more exposed active sites compared to NiFe-LDH and NiFe-La-LDH electrocatalysts [38]. It also discloses an easy electron transfer in the course of OER as well as a potential disassociation of proton and electron transfer [39].

**Figure 8:**
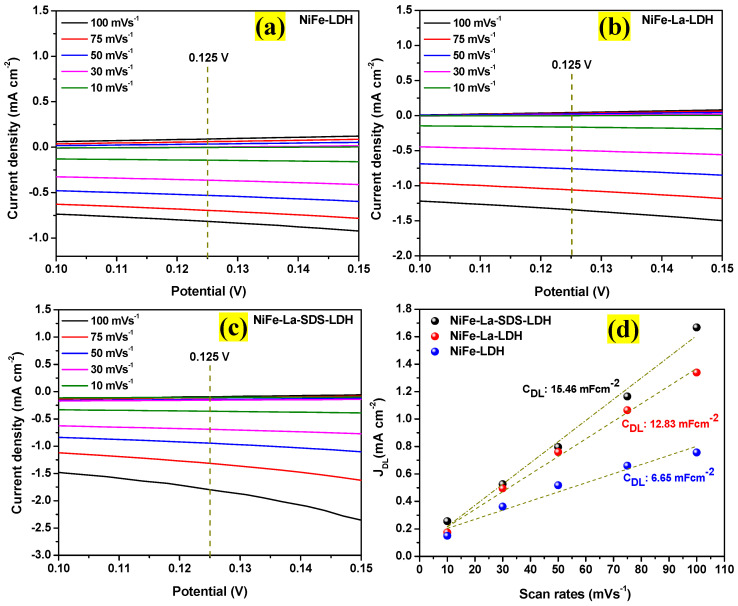
CV curves at non-faradaic zone ranging from 0.10 to 0.15 V at various scan rates (10–100 mVs^−1^) for: (**a**) NiFe-LDH, (**b**) NiFe-La-LDH, and (**c**) NiFe-La-SDS-LDH, and (**d**) non-faradaic charging currents as function of scan rates at the potential of 0.125 V for NiFe LDH, NiFe-La-LDH, and NiFe-La-SDS-LDH.

Subsequently, the electrochemically active surface area (ECSA) was estimated to evaluate the catalytically active sites. From the CV curves of each electrocatalyst recorded at different scan rates (10–100 mVs^−1^), the non-faradaic charging currents were extracted from non-faradaic zones ranging from 0.100 to 0.140 V for all the electrocatalysts as displayed in Figure 8a–c. Afterwards, using Equation (1) [38], the non-faradaic capacitance (*C_Dl_*) for each electrocatalyst was deducted from the slope of the non-faradaic charging current as a function of scan rates at the potential of 0.120 V as shown in Figure 8d. Thus, the deducted non-faradaic capacitances (*C_Dl_*) were to be 6.65 mFcm^−2^, 12.83 mFcm^−2^, and 15.46 mFcm^−2^ for NiFe-LDH, NiFe-La-LDH, and NiFe-La-SDS-LDH electrocatalysts, respectively. Thereafter, using Equation (2) [38], ECSA for NiFe-LDH, NiFe-La-LDH, and NiFe-La-SDS-LDH electrocatalysts were calculated to be 166.25 cm^2^, 320.75 cm^2^, and 386.5 cm^2^, respectively. This indicates that NiFe-La-SDS-LDH electrocatalyst possesses greater OER electrocatalytic activity and bigger ion storage due to its larger ECSA compared to its counterparts [38].

(1) $$J_{DL}=C_{Dl}xSc.rates/A$$

(2) $$ECSA=C_{Dl}/C_{e}$$

where *J_DL_*, *C_Dl_*, A, and *C_e_* represent the non-faradaic charging current, the non-faradic capacitance, the area of the electrode, as well as the unit area capacitance of the electrolyte (0.04 mF/cm^2^ for KOH), respectively [38].

**Figure 9:**
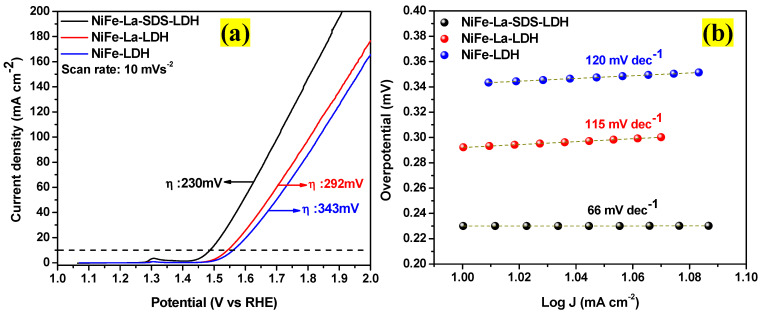
(**a**) iR corrected LSV curves at 10 mA cm^−2^ for NiFe-LDH, NiFe-La-LDH, and NiFe-La-SDS-LDH; (**b**) Tafel slopes of NiFe-LDH, NiFe-La-LDH, and NiFe-La-SDS-LDH.

The OER performance of NiFe-LDH, NiFe-La-LDH, and NiFe-La-SDS-LDH electrocatalysts was further evaluated by the linear sweep voltammetry (LSV) technique. Figure 9a displays the iR-corrected LSV curves for all the electrocatalysts at the scan rate of 10 mVs^−1^ in the potential range of 0 to 1.5 V. The electrochemical activities were iR corrected using Equation (3) [19], while Equation (4) [19] served to estimate the overpotentials (ɳ) for all the electrocatalysts. Thus, it was noticed that NiFe-La-SDS-LDH electrocatalyst exhibited an overpotential of ɳ = 230 mV at a current density of 10 mA cm^−2^ which was lower compared to those of NiFe-La-LDH and NiFe-LDH electrocatalysts at the same current density (ɳ = 292 mV, and 343 mV, respectively). More importantly, Table 2 demonstrates that the overpotential recorded for the NiFe-La-SDS-LDH electrocatalyst was comparable to or even better than previously reported ternary LDHs-based electrocatalysts. Furthermore, using Equation (5), the Tafel slopes for all the electrocatalysts were deducted as disclosed in Figure 9b. The NiFe-La-SDS-LDH electrocatalyst showed a lower Tafel slope of 66 mV dec^−1^ compared to those of NiFe-La-LDH and NiFe-LDH electrocatalysts (115 and 120 mV dec^−1^, respectively). This shows the speedy kinetics of the NiFe-La-SDS-LDH electrocatalyst for OER compared to its counterparts.

(3) $$E_{RHE}=E_{Ag/AgCl}+0.059pH+0.1976$$

(4) $$ɳ=E_{RHE}-1.23$$

(5) $$ɳ=b\log\left(j\right)+a$$

where *E*_*Ag*/*AgCl*_ represents the measured potential versus the reference electrode. The Nernst constant is 0.059 and 0.1976 is the potential of the reference electrode used. ɳ is the overpotential, “*a*” indicates the fitting parameter, while “*b*” is the Tafel slope and *j* the current density. The pH of 1 M KOH was 13.9.

**Figure 10:**
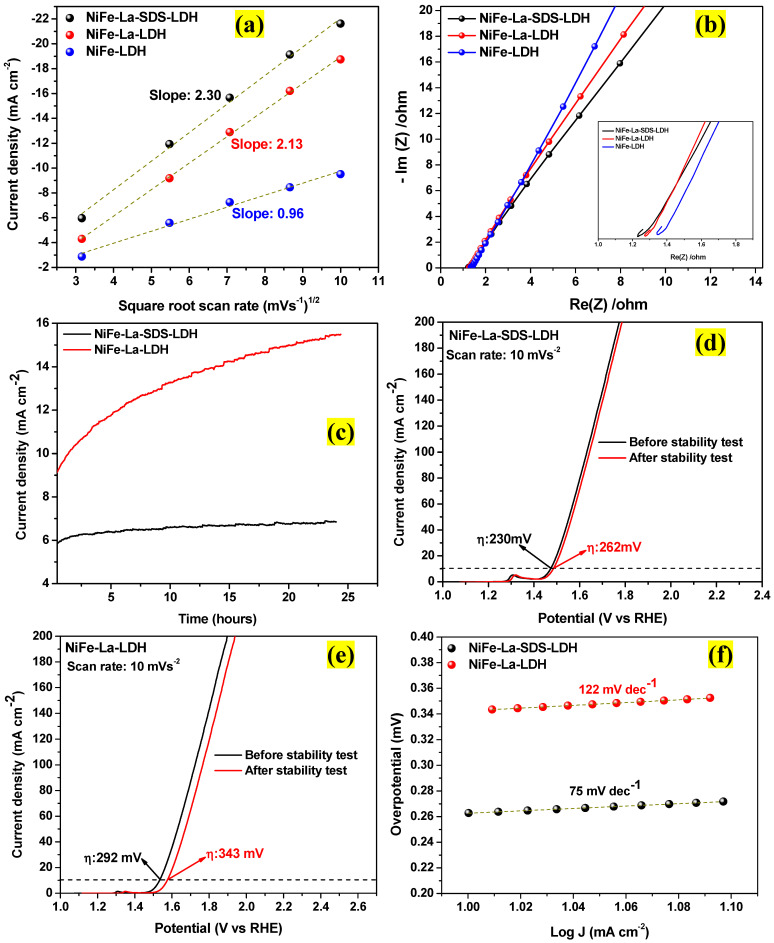
(**a**) plots of the cathodic peak current vs. the square root of the scan rate, (**b**) Nyquist plots for NiFe-LDH, NiFe-La-LDH, and NiFe-La-SDS-LDH, (**c**) chronoamperometry stability (**d**,**e**) iR corrected LSV curves at 10 mA cm^−2^ for NiFe-La-LDH, and NiFe-La-SDS-LDH after stability tests; (**f**) Tafel slopes for NiFe-La-LDH, and NiFe-La-SDS-LDH after stability tests.

Afterwards, the focus was directed to determining the ${OH}^{-}$ diffusion coefficient. It is accepted that the OER mechanism under an alkaline electrolyte includes four proton-coupled electron stages as detailed in Equations (6)–(10) [27]. The first stage is associated with the adsorption of ${OH}^{-}$ on the active site [26]. Technically, the oxygen is released when ${OH}^{-}$ gets absorbed into the active site of the anode. Then, via a series of reactions that initiate intermediates such as *O*, *HOO*, and *OO*, oxygen is released from *OO* [27]. Using the Randles-Sevcik equation which reveals the linear dependence of the peak current as a function of the square root of the scan rate (*υ*^1/2^), the rate of ${OH}^{-}$ diffusion can be determined [26]. Hence, the value of the slope (k), which refers to the ${OH}^{-}$ diffusion, can be deducted from the cathodic peak current as a function of the square root of *υ*^1/2^, using Equation (11) as displayed in Figure 10a [26]. Thereafter, it was observed that NiFe-La-SDS-LDH electrocatalyst possesses a higher (k) value compared to its counterparts (2.30, 2.13, 0.96 for NiFe-La-SDS-LDH, NiFe-La-LDH, and NiFe-LDH electrocatalysts, respectively). This shows that the ${OH}^{-}$ reacts faster with the active sites on NiFe-La-SDS-LDH electrocatalyst than on its counterparts. This also speeds up the electrocatalyst reaction during the *OER* process.

(6) $${OH}_{\left(aq\right)}^{-}+S_{OER}\to HO+e^{-}$$

(7) $${OH}_{\left(aq\right)}^{-}+HO\to O+H_{2}O_{\left(aq\right)}+e^{-}$$

(8) $${OH}_{\left(aq\right)}^{-}+O\to HOO+e^{-}$$

(9) $${OH}_{\left(aq\right)}^{-}+HOO\to OO+H_{2}O_{\left(aq\right)}+e^{-}$$

(10) $$OO\to O_{2(g)}+S_{OER}$$

where $S_{OER}$, $\left(aq\right)$, and (g) represent the active sites on the anode, aqueous, and gas phase, respectively.

(11) $$i=0.446nFAC_{0}{\left(\frac{nF\upsilon D}{RT}\right)}^{1/2}$$

where *n*, *F*, *A*, *C*_0_, *D*, *R*, and *T* are the number of transferred electrons, the Faraday constant, the area of the electrode, the analyte concentration, the diffusion coefficient, the ideal gas constant, and absolute temperature, respectively. However, in the current case, *n*, *A*, *C*_0_, and *υ* values were deducted from *D*, which depends on the slope of the cathodic peak current as a function of the square root of *υ*^1/2^.

Subsequently, the electrochemical impedance spectroscopy (EIS) measurements were done for all the electrocatalysts. Figure 10b shows the Nyquist plots for NiFe-LDH, NiFe-La-LDH, and NiFe-La-SDS-LDH electrocatalysts. The absence of semicircles was noticed in the EIS spectra of all the electrocatalysts. In contrast, linear features in the high-frequency region were observed. This could be attributed to the dispersion of the electrolytic solution [38,47,48]. The inserted image in Figure 10b shows that NiFe-La-SDS-LDH electrocatalyst exhibited lower ionic resistance than its counterparts. This could be ascribed to a smaller charge transfer resistance indicating a faster diffusion process and improved electrical conductivity, which could improve the electrocatalytic performance [38,49,50]. Moreover, the stability tests were carried out via the chronoamperometry technique for both NiFe-La-LDH and NiFe-La-SDS-LDH electrocatalysts using potentials of the onset points. Figure 10c shows the chronoamperometry stability results for NiFe-La-LDH, and NiFe-La-SDS-LDH electrocatalysts measured for 24 h under electrochemical water oxidation. After the stability tests, the OER performances of both electrocatalysts were measured through the LSV technique. It was observed that the NiFe-La-SDS-LDH electrocatalyst exhibited an overpotential of 262 mV at a scan rate of 10 mVs^−1^ which shows a loss of 12% of its OER performance compared to the performance before the stability test. Thereafter, the NiFe-La-LDH electrocatalyst recorded an overpotential of 343 mV at a scan rate of 10 mVs^−1^ showing a loss of 18% of its OER performance compared to the one before the test. Subsequently, the Tafel slopes for both electrocatalysts were calculated after the stability tests to be 75 mV dec^−1^ and 122 mV dec^−1^ for NiFe-La-SDS-LDH and NiFe-La-LDH electrocatalysts, respectively. Comparing the OER performance of both electrocatalysts after 24 h of the stability tests, it is obvious that NiFe-La-SDS-LDH electrocatalyst lost less than NiFe-La-LDH. This could be attributed to its enriched active sites and improved electrical conductivity [51].

**Figure 11:**
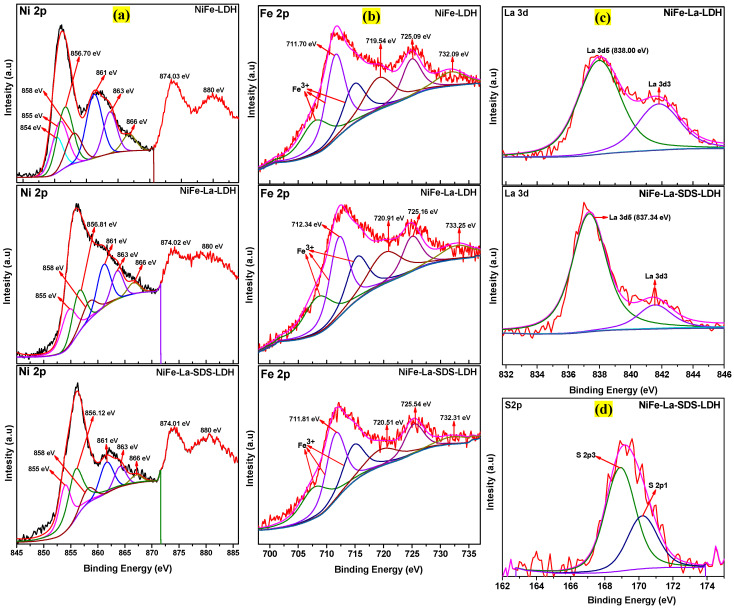
High-resolution spectra of: (**a**) Ni 2p for NiFe-LDH, NiFe-La-LDH, and NiFe-La-SDS-LDH, (**b**) Fe 2p for NiFe-LDH, NiFe-La-LDH, and NiFe-La-SDS-LDH, (**c**) La 3d for NiFe-La-LDH and NiFe-La-SDS-LDH, and (**d**) S 2p for NiFe-La-SDS-LDH.

The X-ray Photoelectron Spectroscopy (XPS) analysis was carried out to understand the improved OER activity of NiFe-La-SDS-LDH electrocatalyst compared to its counterparts. Figure S2a–c shows the XPS survey scan spectra of NiFe-LDH, NiFe-La-LDH, and NiFe-La-SDS-LDH. As it can be noticed, all the expected chemical elements were present including Ni, Fe, O, La, and S, which were in accordance with the EDS results discussed earlier. Furthermore, high-resolution XPS spectra for all the samples were collected. Figure 11a displays the high-resolution spectra of Ni 2p for NiFe-LDH, NiFe-La-LDH, and NiFe-La-SDS-LDH. Firstly, the Ni 3p peaks for all the samples were selected for deconvolution due to La 3d overlapping with Ni 2p. Major peaks with binding energies (BE) of 856.70 eV, 856.81 eV, and 856.12 eV were visible in the Ni 2p spectra of NiFe-LDH, NiFe-La-LDH, and NiFe-La-SDS-LDH, respectively and were ascribed to Ni 2p_3/2_. Afterwards, peaks with BE of 874.03 eV, 874.02 eV, and 874.1 eV were also depicted in the Ni 2p spectra of NiFe-LDH, NiFe-La-LDH, and NiFe-La-SDS-LDH, respectively and were all attributed to Ni 2p_1/2_. Thereafter, peaks corresponding to the shakeup satellites with BE of 861 eV and 880 eV were observed in the Ni 2p spectra of all the samples [2,16,52]. Furthermore, other peaks were also visible in the Ni 2p spectra of NiFe-LDH, NiFe-La-LDH, and NiFe-La-SDS-LDH with BE of 854 eV, 855 eV, 858 eV, 863 eV, and 866 eV. Peaks with BE of 854 eV, 855 eV, and 858 eV could be ascribed to Ni-oxide and hydroxides [53], while peaks with BE of 863 eV and 866 eV corresponded to Ni 2p3. These recorded energy values confirm the N^2+^ or N^3+^ oxidation states [2]. Figure 11b is the high-resolution spectra of Fe 2p for all the samples. Peaks corresponding to Fe 2p_3/2_ and Fe 2p_1/2_ orbital spins were located with BE of 711.70 eV–725.09 eV for NiFe-LDH, 712.34 eV–725.16 eV for NiFe-La-LDH, and 711.81 eV–725.54 eV for NiFe-La-SDS-LDH. These recorded bond energies belonged to Fe(OH)O and were ascribed to Fe^3+^ oxidation state [54]. Generally, the Fe^3+^ oxidation state confirms the formation of LDH [2]; hence, this proves a successful preparation of LDHs. Moreover, peaks attributed to their satellites were also depicted with BE of 719.54 eV–732.09 eV for NiFe-LDH, 720.91 eV–733.25 eV for NiFe-La-LDH, and 720.51 eV–732.31 eV for NiFe-La-SDS-LDH [54]. More importantly, it was noticed that the Fe 2p_3/2_ BE of NiFe-La-LDH shifted positively by 0.64 eV compared to that of NiFe-LDH. Comparing the Fe 2p_3/2_ BE of NiFe-La-SDS-LDH to that of the pristine material, no major change was observed, whereas, a slight shift to lower BE was observed when comparing the Ni 2p_3/2_ BE of NiFe-La-SDS-LDH to that of NiFe-LDH. Moreover, no major change was noticed in the Ni 2p_3/2_ BE of NiFe-La-LDH compared to that of NiFe-LDH. An obvious shifting of La 3d5 BE by 0.66 eV towards lower BE was noticed in the high-resolution spectrum of La 3d for NiFe-La-SDS-LDH compared to that of NiFe-La-LDH as displayed in Figure 11c. It is known that the position of a BE for a particular element is linked to its local chemical environment as well as the oxidation state. This explains that La has modulated the chemical environment as well as the electronic structure of Fe ions and slightly regulated that of Ni ions, resulting in improving the OER performance compared to NiFe-LDH [26]. However, after incorporating SDS, not only was the electronic structure of Ni and Fe ions modulated but that of La was also affected. This could be attributed to the presence of sulphur generated by SDS (see Figure 11d) which has enriched the reaction sites of NiFe-La-SDS-LDH, resulting in an increase in its electrocatalytic performance compared to its counterparts. This has yielded a superior OER activity compared to that of NiFe-LDH and NiFe-La-LDH. Figure S3a–c shows the high-resolution spectra of O 1 s for all the samples. Mean peaks with BE of 529.6 eV, 531.1 eV, 532.9 eV, and 535.5 eV were depicted in the O 1 s spectrum of NiFe-LDH. While others with BE of 530.2 eV, 531.5 eV, 533.2 eV, and 535.5 eV were in that of NiFe-La-LDH. Similar peaks were also observed in the O 1 s spectrum of NiFe-La-SDS-LDH except for the peaks with BE of 535.5 eV. Generally, peaks with BE ranging from 529.6 eV to 530 eV are ascribed to the lattice oxygen [55], while the peak with BE of 531 eV belongs to the hydroxyl group attached to the metal-oxygen (M-O) [56]. Peaks with BE ranging from 532 eV to 533 eV are usually adsorbed organic compounds binding through M-O interactions and water molecules located on the surface of a metal [57,58,59,60]. More often, the binding strength of water molecules bound to the surface of a metal is enhanced by the presence of the hydroxyl groups, resulting in O 1 s peaks with BE of 531 eV and 532 eV [59,60]. Finally, the peak with BE of 535 eV is ascribed to the adsorbed gas phase water [58,60]. There is a similarity in the O 1 s spectra of NiFe-LDH and NiFe-La-LDH, except for the intensities of peaks which could result from the change in their intermolecular hydrogen bond network due to La [60]. In contrast, the O 1 s spectrum of NiFe-La-SDS-LDH is completely different from those of its counterparts showing that the electronic structures of the three samples were not alike.

## 4. Conclusions

This study presents a promising approach for modulating the electronic structures of the three cations involved in the synthesis of ternary LDHs. The proposed method suggested incorporating sodium dodecyl sulfate (SDS) into NiFe-La-LDH by intercalating it within the interlayer. Fourier Transform Infrared (FT-IR) results confirm the successful intercalation of SDS, which led to an expansion of the interlayer. This expansion, as indicated by the X-ray Diffraction (XRD) results, is beneficial for enhancing electrical conductivity. Furthermore, Energy Dispersive Spectroscopy (EDS) and X-ray Photoelectron Spectroscopy (XPS) confirmed the presence of sulphur generated by the SDS, which enabled the modulation of the electronic structures of all three cations, as demonstrated by the XPS results. As a result, the NiFe-La-SDS-LDH electrocatalyst showed improvement in several parameters related to its performance during the OER process. These enhancements include better electron transfer, increased kinetics, improved reaction speed at active sites, higher electrical conductivity, enhanced electrocatalytic activity, and greater ion storage. Consequently, this electrocatalyst demonstrates excellent OER performance compared to its counterparts. Specifically, the NiFe-La-SDS-LDH electrocatalyst achieved a lower overpotential of 230 mV at a current density of 10 mA cm⁻^2^, outperforming its counterparts at the same current density (NiFe-La-LDH had an overpotential of 292 mV, and NiFe-LDH had 343 mV). Additionally, its Tafel slope was calculated to be lower at 66 mV dec⁻^1^, compared to 115 mV dec⁻^1^ for NiFe-La-LDH and 120 mV dec⁻^1^ for NiFe-LDH. Electrochemical impedance spectroscopy results indicated that NiFe-La-SDS-LDH has a lower resistance compared to its counterparts. Furthermore, the specific surface area of NiFe-La-SDS-LDH increased by 2.5 times than those of NiFe-La-LDH and NiFe-LDH. This approach holds promise and offers several advantages: 1. The interlayer can be further expanded by using different doping ratios of SDS. It is known that a larger interlayer contributes to better performance. 2. The generated sulphur can enrich the active sites, leading to improved OER performance.

## Figures and Tables

**Table 1:** Textural Properties for NiFe-LDH, NiFe-La-LDH, and NiFe-La-SDS-LDH.

Samples	Surface Area (m^2^g^−1^)	Pore Size (nm)	Pore Volume (cm^2^g^−1^)
NiFe-LDH	143.69	9.20	0.33
NiFe-La-LDH	184.95	7.78	0.36
NiFe-La-SDS-LDH	417.15	10.50	1.09

**Table 2:** Comparison of OER performance of NiFe-La-SDS-LDH electrocatalyst with previously reported ternary LDHs-based electrocatalysts.

Electrocatalysts	Overpotential	Current Density	References
NiFeCo-LDH	253 mV	10 mA cm^−2^	[40]
CoCuFe-LDH/Graphene	350 mV	10 mA cm^−2^	[41]
NiFeCo-LDH	249 mV	10 mA cm^−2^	[19]
NiCoFe-LDH/CFC	240 mV	10 mA cm^−2^	[42]
Ternary NiCoMo	270 mV	10 mA cm^−2^	[21]
NiAlFe-LDH	236 mV	0.7 mA cm^−2^	[43]
NiCoRu-LDH	270 mV	100 mA cm^−2^	[44]
NiFeMn-LDH	338 mV	10 mA cm^−2^	[45]
NiFeCr@3D V2C-MX	410 mV	200 mA cm^−2^	[46]
NiFeZn-LDH	236 mV	50 mA cm^−2^	[22]
**NiFe-La-SDS-LDH**	**230 mV**	**10 mA cm^−2^**	**This work**

## Data Availability

The data presented in this study are available on request from the corresponding author.

## Supplementary Materials

The following supporting information can be downloaded at: https://www.mdpi.com/article/10.3390/nano15030177/s1, Figure S1a–c: Mapping images for NiFe-LDH, NiFe-La-LDH, NiFe-La-SDS-LDH; Figure S2a–c: XPS survey scan spectra for NiFe-LDH, NiFe-La-LDH, NiFe-La-SDS-LDH; Figure S3a–c: O 1 s high-resolution for NiFe-LDH, NiFe-La-LDH, NiFe-La-SDS-LDH.
