# Supplementary material for: Improving the Oxygen Evolution Reaction Performance of Ternary Layered Double Hydroxides by Tuning All Three Cations’ Electronic Structures
Source: Nanomaterials (Basel). 2025 Jan 23;15(3):177. doi: 10.3390/nano15030177 (PMC11820138; doi:10.3390/nano15030177)
Supplement: Supplementary file 1 [file nanomaterials-15-00177-s001.zip › nanomaterials-3448540-supplementary.pdf]

Supporting Information

Improving the Oxygen Evolution Reaction Performance of the Ternary Layered Double Hydroxides by Tuning all three cations' Electronic Structures

Gayi Nyongombe[1,3]*, Malik Maaza[2], Mohamed Siaj[3], M.S. Dhlamini[1]*

[1]Department of Physics, School of Science, CSET, University of South Africa, Private Bag X6, Florida, 1710, Science Campus, Christiaan de Wet and Pioneer Avenue, Florida Park, Johannesburg, South Africa.

[2] UNESCO-UNISA Africa Chair in Nanosciences/Nanotechnology, College of Graduate Studies, University of South Africa, Muckleneuk Ridge, P.O. Box 392, Pretoria, South Africa.

[3] NanoQAM Center, Department of Chemistry, University of Quebec at Montreal, Montreal, QC, H3C3P8, Canada. E-mail: **siaj.mohamed@uqam.ca**

*E-mail: gayinyongombe@gmail.com, dhlamms@unisa.ac.za

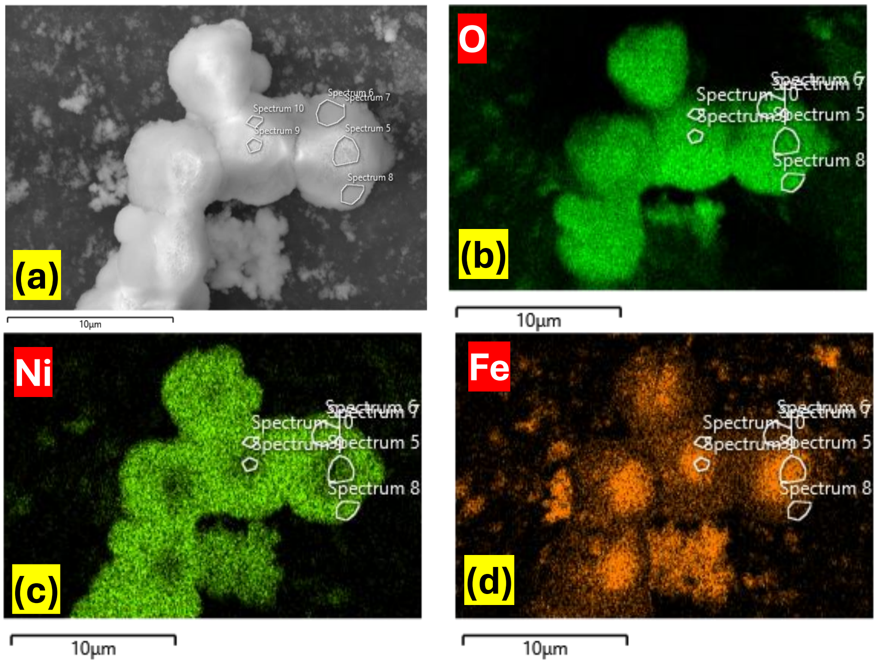

Fig.S1a: c: (a) NiFe-LDH,

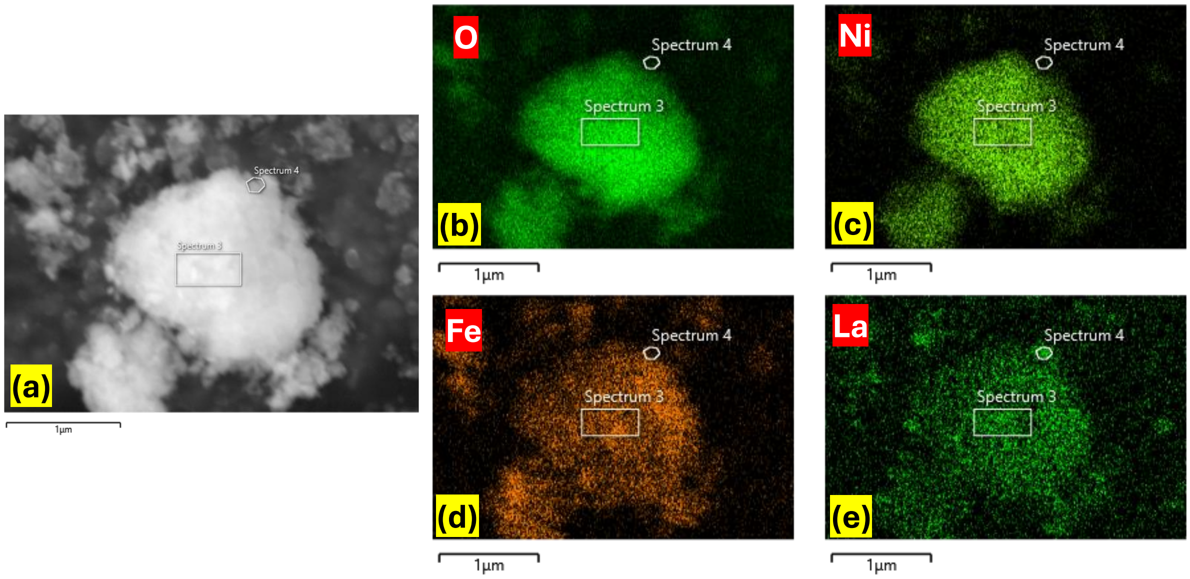

Fig.S1b: Mapping image for: (a) NiFe-La-LDH,

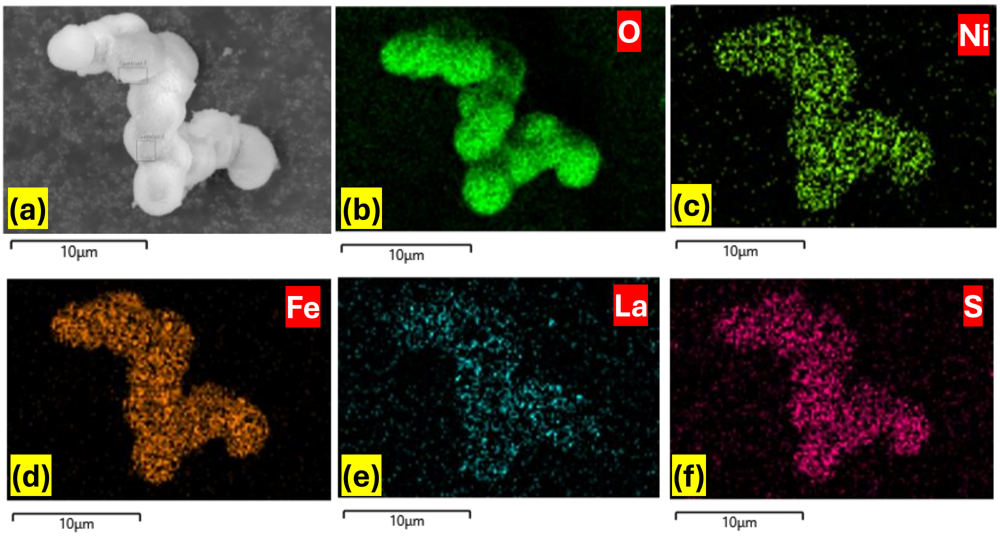

Fig.S1c: Mapping image for: (a) NiFe-La-SDS-LDH,

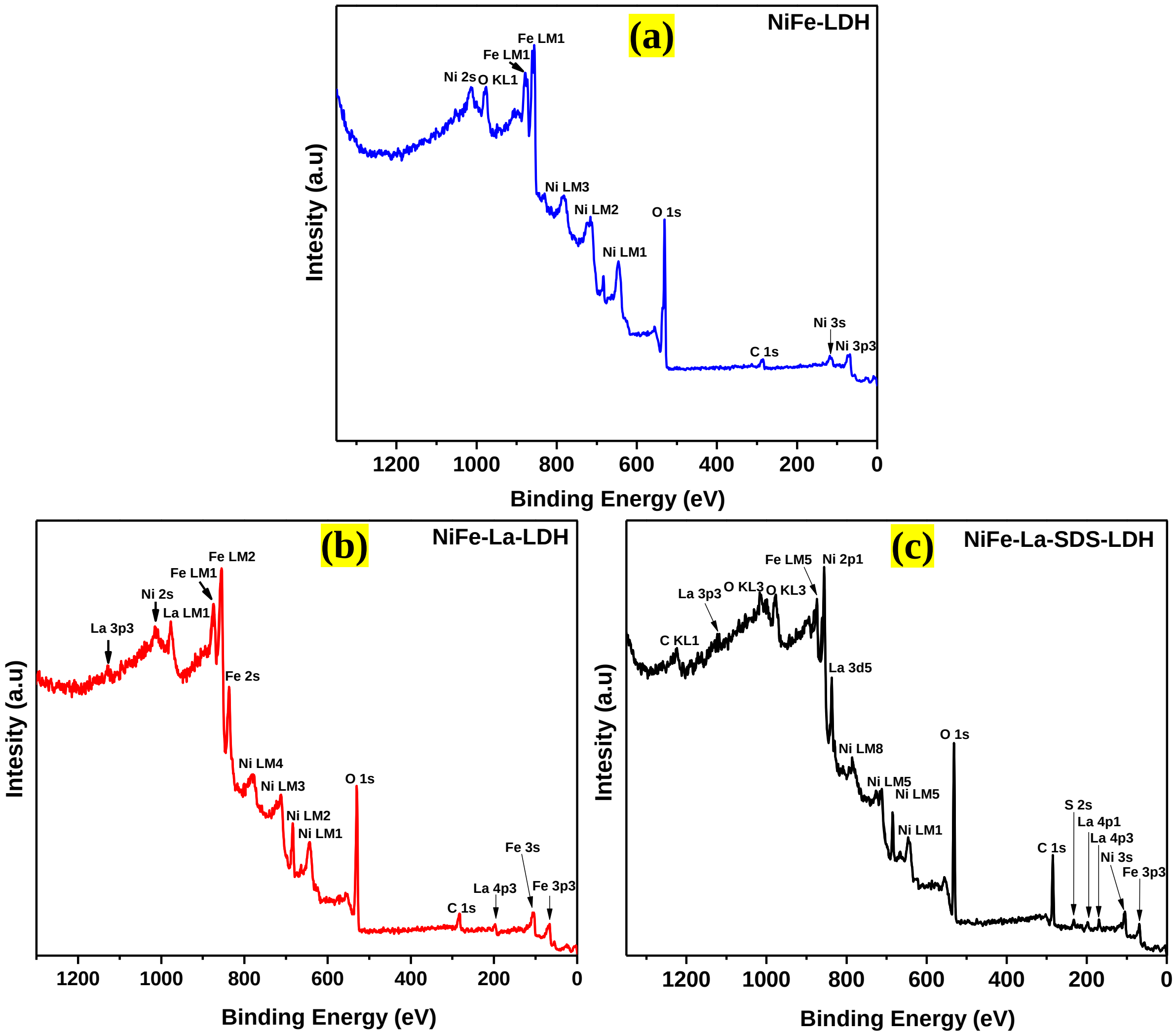

Fig.S2: XPS survey scan spectra for: (a) NiFe-LDH, (b) NiFe-La-LDH, and (c) NiFe-La-SDS-LDH.

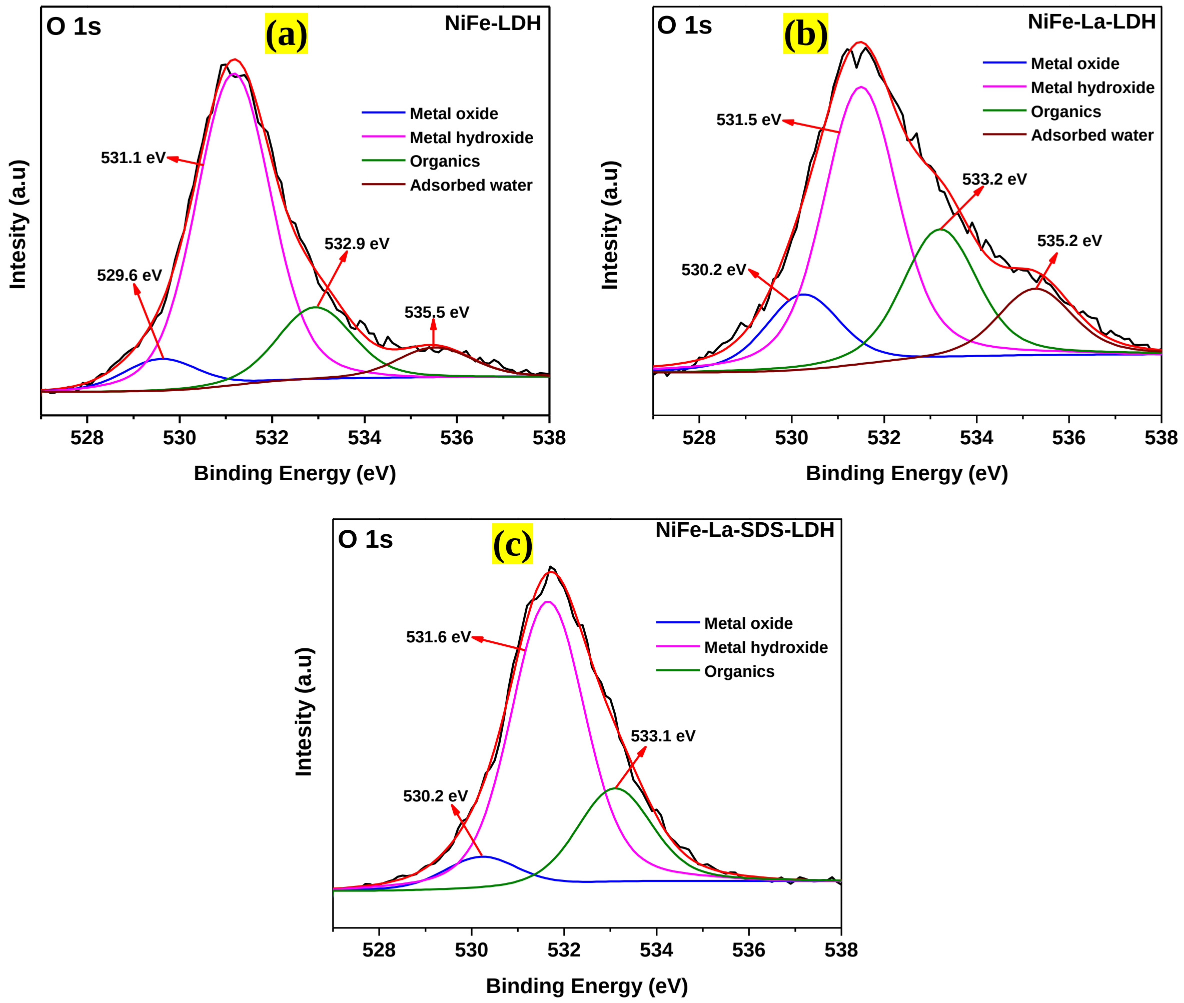

Fig.S3: O 1s high-resolution of: (a) NiFe-LDH, (b) NiFe-La-LDH, and (c) NiFe-La-SDS-LDH.
